# Acute consumption of a branched chain amino acid and vitamin B-6 containing sports drink does not improve multiple sprint exercise performance, but increases post-exercise blood glucose

**DOI:** 10.3389/fnut.2023.1266422

**Published:** 2023-12-05

**Authors:** Saro D. Farra

**Affiliations:** Faculty of Applied Health and Community Studies, Sheridan College, Brampton, ON, Canada

**Keywords:** BioSteel, branched chain amino acids, vitamin B, multiple sprint exercise, blood glucose, cycling, nutritional supplements, sports drinks

## Abstract

**Purpose:**

The aim of this study was to investigate the ergogenicity of BioSteel High Performance Sports Drink (B-HPSD), a commercially available branched chain amino acid (BCAA) and vitamin B-6 (VitB-6) supplement, on multiple sprint exercise (MSE).

**Methods:**

Eleven experienced cyclists completed two MSE trials in counterbalanced order, after ingesting either B-HPSD (2,256 mg of BCAA, 300 mcg of VitB-6) or placebo (PLA). The MSE protocol consisted of five maximal effort 1 km sprints on a cycle ergometer separated by 2 min of active recovery. Power output (PO) was continuously measured throughout the cycling protocol. Heart rate (HR) and ratings of perceived exertion (RPE) were monitored following each sprint. Capillary blood samples were collected and analyzed for lactate and glucose before and 2 min post-trial. Cognitive function was assessed before and 15 min after the exercise protocol.

**Results:**

The PO maintained during each 1 km sprint decreased throughout the protocol (*p* < 0.05), but the change in PO was similar between conditions. Post-exercise blood glucose was elevated after consuming B-HPSD but not PLA (*p* < 0.05). Blood lactate (*p* < 0.05), HR (*p* < 0.05) and RPE (*p* < 0.05) increased throughout the trials, however no differences were observed between conditions. Cognitive performance improved after exercise (*p* < 0.05), but the change was similar between conditions.

**Conclusion:**

These results demonstrate that acute B-HPSD consumption does not have an ergogenic effect on MSE performance. However, ingestion of B-HPSD increased post-exercise blood glucose concentration when compared to PLA.

## 1 Introduction

Exercise-induced fatigue during multiple sprint exercise (MSE), is a task-dependent, transient decrease in the ability to achieve maximal speed or peak power output (PO) over successive sprints. The ‘all-out’ nature of MSE stresses metabolic and neural pathways, compromising the ability to repeat sprint efforts (1). The effects of various nutritional supplements, such as carbohydrate (2–5), creatine (6–8), as well as caffeine and caffeine-containing energy drinks (9–15) on MSE have been extensively investigated. However, the ergogenic effects of branched chain amino acids (BCAA) on MSE have received little attention (16).

During MSE, active muscles progressively shift from anaerobic glycolysis/glycogenolysis to aerobic metabolism (1, 17) and the oxidation of various non-glycolytic fuel sources, including pyruvate (18), intramuscular lipids (19) and BCAA (20). Esbjörnsson et al. (20) observed decreasing plasma BCAA and increasing muscle ammonia (NH_3_) concentrations over three 30 s ‘all-out’ efforts on a cycle ergometer; suggesting elevated breakdown of BCAA in skeletal muscle, as glutamate derived from BCAA transamination (21) reacts with NAD+ to form α-ketoglutarate and NH_3_ (22). In addition, direct evidence illustrating elevated rates of BCAA catabolism was confirmed when α-ketoisocaproic acid, the branched chain keto acid (BCKA) associated with leucine transamination, increased in skeletal muscle following a bout of high-intensity exercise (23). BCKAs are further degraded by BCKA dehydrogenase, the rate limiting enzyme in BCAA catabolism (24). BCKA dehydrogenase is inactive at rest but is activated during exercise in direct proportion to exercise intensity (25, 26). This activation leads to increased production of acetyl-CoA and succinyl-CoA, which enter the Krebs cycle and generate ATP via oxidative phosphorylation (27). Given the low resting activity of BCKA dehydrogenase and the capacity of exercise to active this rate-limiting enzyme, the increase in BCAA oxidation may progressively supply energy during MSE with sequential efforts. Therefore, when active muscles transition away from glycolytic substrates during MSE, exogenous BCAAs can potentially serve as an alternative energy source. However, compared to other substrates, their contribution to ATP production may be limited (28).

Despite extensive research on the influence of BCAA on prolonged aerobic exercise lasting over 60 min (29–35), limited research exists regarding their ergogenic benefits for MSE. The existing studies present contradictory findings, as Pitkänen et al. (36) found administering 200 mg/kg body weight BCAA alone had no effect on MSE, while Hsueh et al. (16) observed improved MSE performance when supplementing with 85 mg/kg body weight BCAA in combination with citrulline (CIT) and arginine (ARG). The conflicting results may be attributed to various factors associated with varying BCAA dosage and the co-ingestion of BCAA with other nutrients. Accordingly, manufacturers have been creating supplements that blend BCAA with other ingredients, to enhance the effectiveness of BCAA in attenuating exercise-induced fatigue. For example, BioSteel High Performance Sports Drink (B-HPSD) has become a popular supplement for both professional and amateur athletes. B-HPSD is a sugar-and caffeine-free beverage that provides 2,256 mg of BCAA in combination with 300 mcg of VitB-6 and other cofactors per serving. VitB-6 plays a critical role in the transamination of BCAA (37, 38), as deficiencies in this vitamin can impair the breakdown of BCAA. It has been reported that 5 to 60% of athletes have poor VitB-6 levels, as exercise increases the turnover and excretion of this vitamin (39). While the individual concentrations of BCAA (40) and VitB-6 (41) in B-HPSD may be insufficient to produce an ergogenic effect, such a blend of ingredients may enhance the utilization of BCAA as a fuel source. However, the ergogenicity of B-HPSD has yet to be assessed in the published literature.

Therefore, the primary purpose of this study was to examine whether acute B-HPSD consumption improves MSE performance. In this investigation, the influence of B-HPSD was evaluated in real-world conditions where participants consumed their normal diet, and without any additional measurements during exercise to avoid disturbing the participants’ performance ability. As such, this study will only be able to elucidate if, and not how, B-HPSD affects MSE performance.

## 2 Methods

### 2.1 Study design

This investigation utilized a double-blind, placebo-controlled, repeated-measures design with two experimental trials completed in random order: B-HPSD and placebo (PLA). The trials were separated by at least 72 h and were completed at the same time of day (within ±1 h), under consistent laboratory conditions. Participants avoided ingesting any food or drink (besides water) for 2 h, avoided caffeine for 12 h, and avoided performing any vigorous exercise for 24 h before any testing session. Each participant maintained an identical nutritional intake 24 h before each experimental trial, which they verbally confirmed prior to starting the exercise protocol. These controls were implemented to standardize conditions between trials and to ensure participants exercised in a fed state, which is common practice for most athletes.

### 2.2 Participants

Eleven apparently healthy subjects volunteered to participate in this investigation (Table 1). Participants were experienced recreational or competitive cyclists. This sample size would detect a treatment at the 0.05 level, with a power of 0.84 assuming a medium effect size (*η*^2^ = 0.06) in the main outcome variable, PO during sprint cycling (G*Power, v.3.1.9.3, Germany). Participants were free of food/drug allergies, conditions that can be aggravated by exercise, as well as metabolic, cardiovascular, and respiratory disorders.

**Table 1:** Physical and physiological subject characteristics.

	*n* (#)	Age (y)	Height (cm)	Weight (kg)	Body Fat (%)	P_max_ (W)	V̇O_2peak_ (ml•kg^−1^•min^−1^)
Male	8	24 ± 5	181 ± 7	82 ± 8	13 ± 4	311 ± 73	49 ± 10
Female	3	28 ± 3	168 ± 4	72 ± 12	27 ± 4	208 ± 29	38 ± 9
Group	11	25 ± 5	178 ± 9	79 ± 11	17 ± 8	283 ± 79	46 ± 11

P_max_ (Final stage completed during V̇O_2peak_ test).

Participants were not supplementing with BCAA prior to starting the study and were also not taking any prescription medications. All female subjects reported the occurrence of monthly menstrual cycles with a consistent duration that ranged between 26 and 30 days for the last 6 months. Female participants were not using oral contraceptives and completed the two experimental trials during the first 7 days of their menstrual cycle, likely during the follicular phase.

Prior to their participation, subjects were informed of the risks associated with the study and a written informed consent was obtained. This study was conducted in accordance with the Declaration of Helsinki and approved by the Research Ethics Board of Sheridan College (SREB Protocol # 2019–02–001-006).

### 2.3 Test protocol

**Figure 1:**
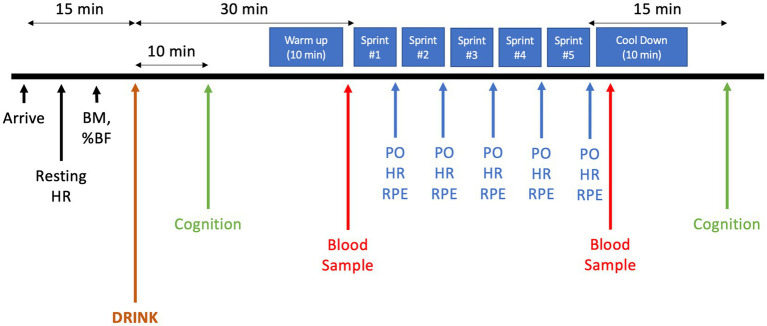
MSE Protocol. HR, Heart rate; BM, Body mass; %BF, Body Fat percentage; PO, Power output; RPE, Ratings of perceived exertion.

Participants reported to the laboratory on four occasions. During the first visit, standing height, body mass, and body composition were measured using a stadiometer, scale, and a hand-held bioelectrical impedance device (Model HBF-306BL, Omron, United States) respectively. Peak oxygen consumption (V̇O_2peak_) was also measured. During the second visit, participants were familiarized with all experimental testing procedures. In the two remaining experimental sessions, measures of resting heart rate (HR) and body mass were obtained upon arrival. Participants were then given 10 min to consume 355 mL of one experimental drink. 30 min after consuming the beverage, participants started the MSE protocol, as previous research demonstrated that plasma BCAA concentrations peak after 30–40 min after ingestion (42). The MSE protocol consisted of five 1 km maximal effort sprints separated by 2 min of active recovery. During the 2 min period immediately preceding each of the five sprints, participants were asked to cycle at an RPE of 3 on Borg’s 10-point scale, with the final stage of the V̇O_2peak_ test as their anchor for what would constitute a “10”; effort was voluntarily adjusted with fatigue, such that subjective sensations of whole-body exertion remained steady at a target level of 3. Participants remained seated throughout the protocol. Participants were informed about the distance remaining during each 1 km sprint and time remaining in the recovery period only. No other visual or verbal feedback about exercise intensity or elapsed time was provided. Changes in PO were continuously measured throughout the trials. HR and ratings of perceived exertion (RPE) were recorded immediately following each sprint. Two capillary blood samples were drawn (pre-and 2 min post-trial) and analyzed for glucose and lactate. A test of cognitive function (Modified Flanker Task) was administered immediately after consuming the experimental drink and again 15 min after completing the MSE protocol (Figure 1).

### 2.4 V̇O_2peak_

Participants completed a graded exercise test to exhaustion on a cycle ergometer to measure V̇O_2peak_. The initial workload of the test was set at 90 W and increased in a step-wise fashion by 30 W every 3 min. The test was terminated when (1) the participant was not able to maintain their cadence within 20 RPM of their self-selected target for more than 30 s; or (2) the participant reached volitional fatigue (43). During the test protocol, expired gas was collected and V̇O_2peak_ was measured using an automated gas analyzer (TrueOne 2,400, ParvoMed, United States). V̇O_2peak_ was defined as the highest 30 s sample collected.

### 2.5 Test drink preparation

**Figure 2:**
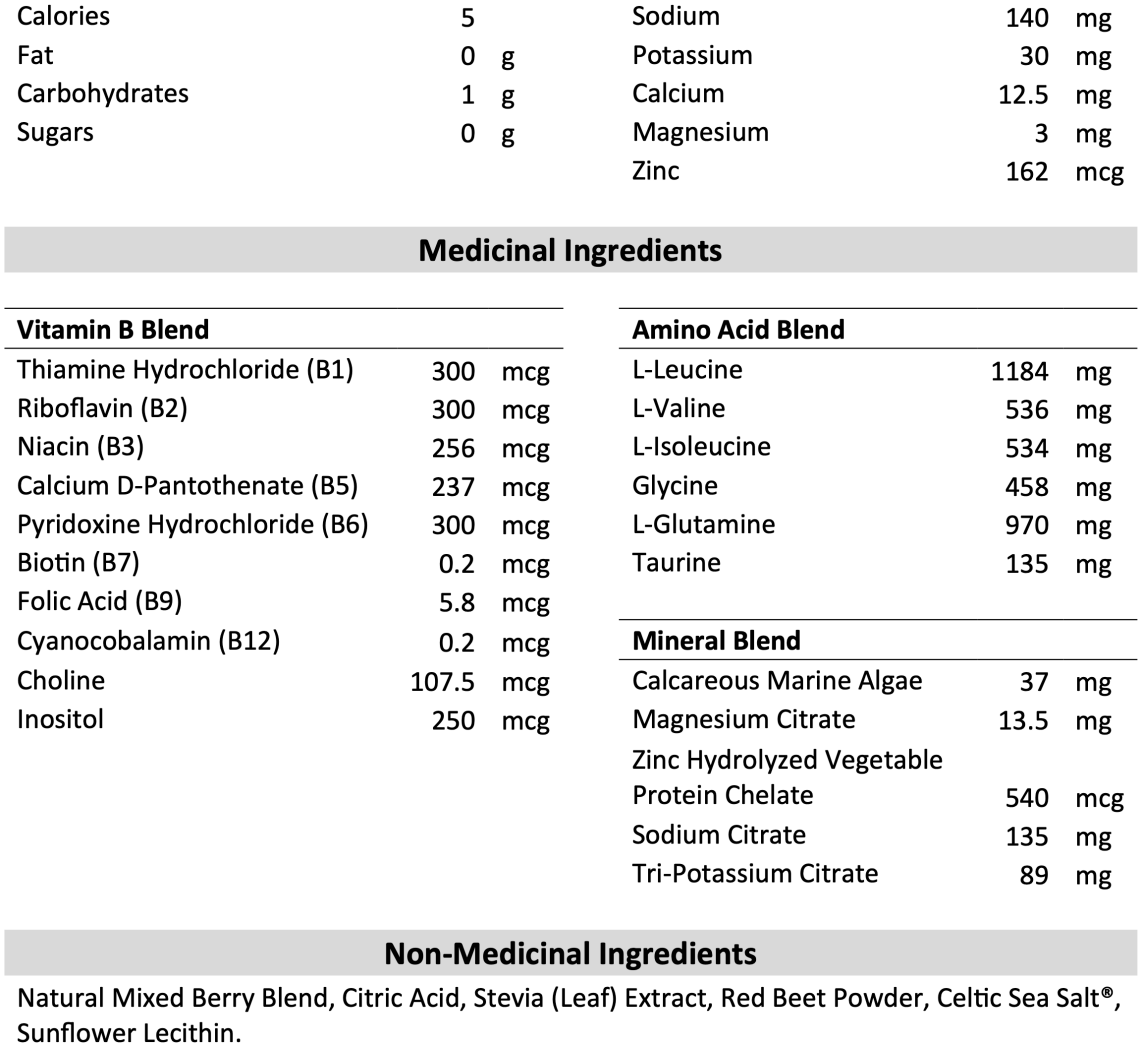
Composition of BioSteel High Performance Sports Drink per serving (7g). Adapted from the Nutrition Label of BioSteel High Performance Sports Drink (2019).

B-HPSD and PLA were obtained in powder form from our industry partner, BioSteel Sport Nutrition Inc. (Toronto, Canada). The composition of B-HPSD is shown in Figure 2. PLA consisted of all non-medicinal ingredients with the addition of 200 mg of silicone dioxide per serving. Drinks were prepared fresh before each session by a member of the research team not responsible for data collection or analysis. According to the manufacturer’s instructions, one serving of B-HPSD (7 g) or PLA (2.75 g) was mixed with 355 mL of water. This amount of PLA provided an equal amount of non-medical ingredients compared to the B-HPSD treatment. Test drinks were provided in opaque bottles to blind participants to the various conditions. Participants has access to water *ad libitum* during the MSE protocol.

### 2.6 Data acquisition

#### 2.6.1 Cycling exercise

All cycling exercise was performed on an electronically braked cycle ergometer (Excalibur Sport, Lode, Groningen, Netherlands) sampling PO at 5 Hz. Participants used the same footwear and pedals for every session. Bike set-up for each subject was recorded in the first session so that it can be subsequently reproduced for the remaining tests. During the V̇O_2peak_ test, the ergometer operated in hyperbolic mode, maintaining the target PO despite changes in cadence. Pedaling rate (RPM), elapsed time, HR, and PO were displayed during the test. Performance was defined as the Final Stage completed and the corresponding V̇O_2peak_. During the MSE protocol, the ergometer operated in isokinetic mode, maintaining a pre-selected RPM despite changes in effort. Participants self-selected their target RPM during the first experimental trial and were required to complete the subsequent trial at that cadence. During all cycling exercise, PO was continuously recorded. PO was averaged over each 1 km sprint. In the 2 min period immediately preceding each sprint, participants could freely adjust their work intensity by modifying their level of force generation to maintain their target of 3. PO was averaged over each 2 min period.

#### 2.6.2 Heart rate and RPE

HR was measured using a heart rate monitor (Polar Electro, Finland) attached around the participant’s chest. RPE was recorded using the Borg 10-point scale (44). This scale was used to quantify perceived effort during exercise. Participants rated their perceived level of physical exertion between 0 = “Rest” and 10 = “Maximal.”

#### 2.6.3 Biochemical measurements

Capillary blood samples were obtained from the participants’ right hand under aseptic conditions. Samples were analyzed for lactate (Lactate Plus, Nova Biomedical, United States) and glucose (Accu-Chek Aviva, Roche, Switzerland).

#### 2.6.4 Modified Flanker Task

The Modified Flanker Task included 100 individual trials. In each trial, five arrows were presented linearly on an iPad screen (BrainBaseline Lab, Digital Artifacts LLC, Iowa). The center arrow was either flanked with arrows pointing in the same direction (congruent) or with arrows pointing in the opposite direction (incongruent). Participants were instructed to press a button on either side of the screen that corresponded to the direction the center arrow was pointing. Response time (RT) and accuracy were measured for 50 congruent and 50 incongruent tasks. Data were analyzed as an overall score out of 100 trials.

### 2.7 Statistical analysis

Results are presented as mean values ± standard deviation. Differences were considered significant when *p* < 0.05. The absolute change in blood glucose was analyzed using a paired t-test. A two-way repeated measures analysis of variance (DRINK x TIME) was performed to evaluate differences in all other variables. When the assumption of sphericity was violated, a Greenhouse–Geisser correction was used. Significant main effects were followed up with pairwise comparisons and significant interaction effects were followed up with paired t-tests. The *p*-values reported for all follow up test were adjusted using a Bonferroni correction. All statistics were calculated using SPSS software (Version 26 for Mac, IBM).

## 3 Results

### 3.1 Power output

There was a main effect of TIME on PO during MSE (*F* = 18.4; *p* = 0.002). PO significantly decreased with subsequent sprints, but the change in PO was similar between DRINKS (Table 2).

**Table 2:** Change in mean power output (W) sustained over each 1 km sprint.

	Sprint				
	1	2	3*	4**	5***
Placebo	643 ± 270	612 ± 276	562 ± 271	504 ± 264	482 ± 263
B-HPSD	643 ± 267	614 ± 274	553 ± 264	500 ± 260	480 ± 261

*Significantly different than Sprint 1 (*p* = 0.02). **Significantly different than Sprint 2 (*p* = 0.019). ***Significantly different than Sprint 3 (*p* = 0.027).

No main (DRINK, *p* = 0.192; TIME, *p* = 0.127) or interaction (DRINK x TIME; *p* = 0.383) effects were detected for the self-selected PO generated while cycling at an RPE of 3 on Borg’s 10-pt scale during the five 2-min periods immediately preceding each sprint (Table 3).

**Table 3:** Change in mean power output (W) sustained over the 2 min period immediately preceding each 1 km sprint.

	Sprint				
	1	2	3	4	5
Placebo	73 ± 35	70 ± 30	63 ± 28	57 ± 33	56 ± 31
B-HPSD	84 ± 45	74 ± 32	68 ± 31	61 ± 32	54 ± 32

### 3.2 Heart rate and RPE

There was a main effect of TIME on HR following each sprint (*F* = 114.6; *p* < 0.001). HR significantly increased throughout the exercise protocol, but the change in HR was similar between DRINKS (Table 4).

**Table 4:** Heart rate (bpm) following each 1 km sprint.

	Sprint				
	1	2	3*	4**	5***
Placebo	161 ± 10	168 ± 9	171 ± 8	172 ± 8	174 ± 7
B-HPSD	162 ± 11	168 ± 10	172 ± 9	174 ± 8	175 ± 8

*Significantly different than Sprint 1 (*p* < 0.001). **Significantly different than Sprint 2 (*p* < 0.001). ***Significantly different than Sprint 3 (*p* < 0.001).

There was a main effect of TIME on RPE following each sprint (*F* = 26.3; *p* < 0.001). RPE significantly increased throughout the exercise protocol, but the change in RPE was similar between DRINKS (Table 5).

**Table 5:** Rating of perceived exertion following each 1 km sprint.

	Sprint				
	1	2	3*	4**	5***
Placebo	6 ± 2	8 ± 2	8 ± 2	9 ± 1	10 ± 1
B-HPSD	6 ± 2	7 ± 2	8 ± 1	9 ± 1	10 ± 1

*Significantly different than Sprint 1 (*p* = 0.002). **Significantly different than Sprint 2 (*p* = 0.023). ***Significantly different than Sprint 3 (*p* = 0.036).

### 3.3 Biochemistry

There was a main effect of TIME on blood lactate during the MSE protocol (*F* = 787.8; *p* < 0.001). Blood lactate significantly increased post-exercise, but the change in lactate was similar between DRINKS (Table 6).

**Table 6:** Change in blood lactate (mmol) pre-and post-MSE.

	Pre	Post*
Placebo	1.0 ± 0.4	13.1 ± 1.9
B-HPSD	1.4 ± 0.7	13.4 ± 1.3

*Significantly different than Pre (*p* < 0.001).

**Figure 3:**
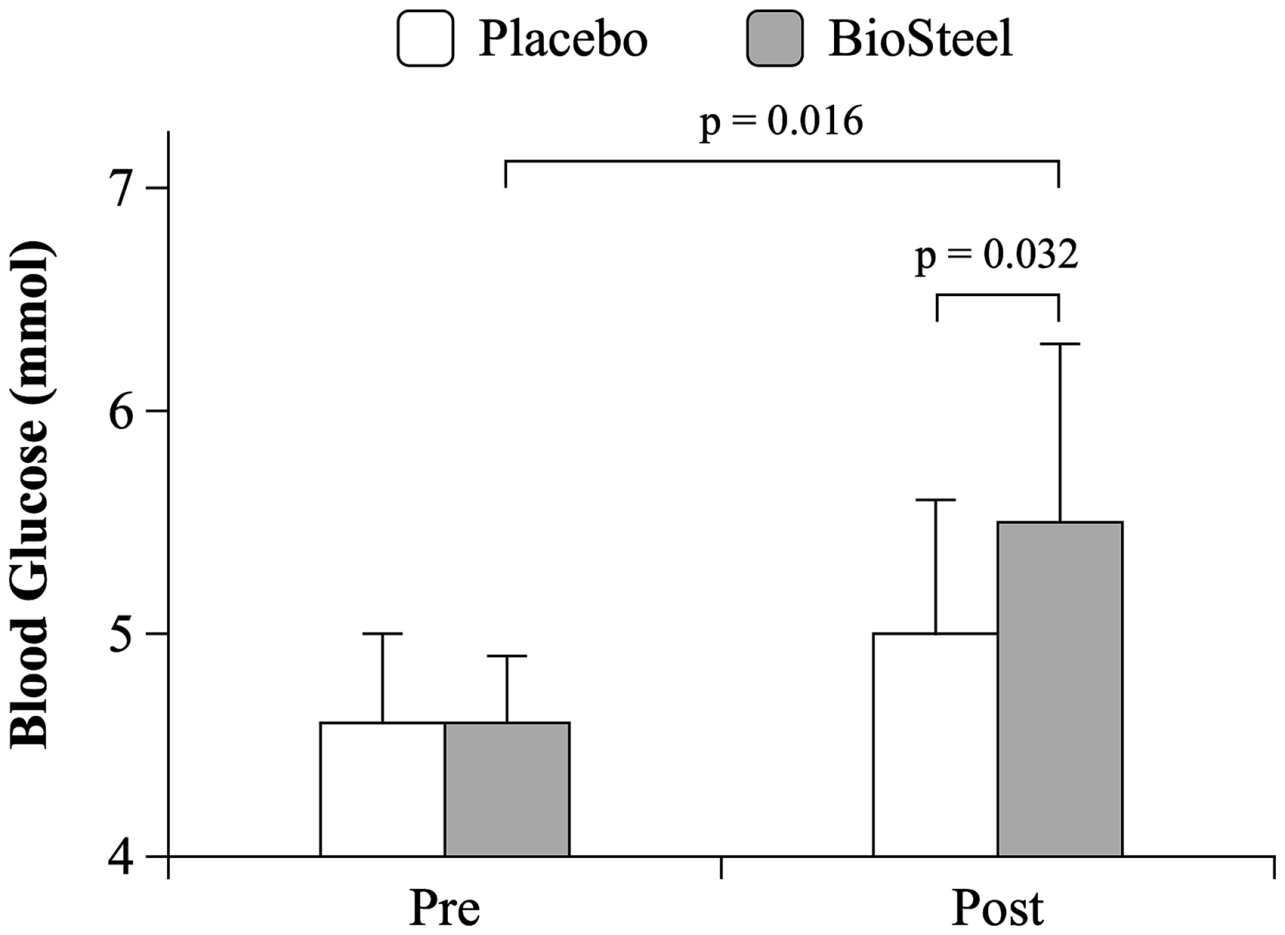
Blood glucose response pre-and post-MSE for PLA and B-HPSD.

**Figure 4:**
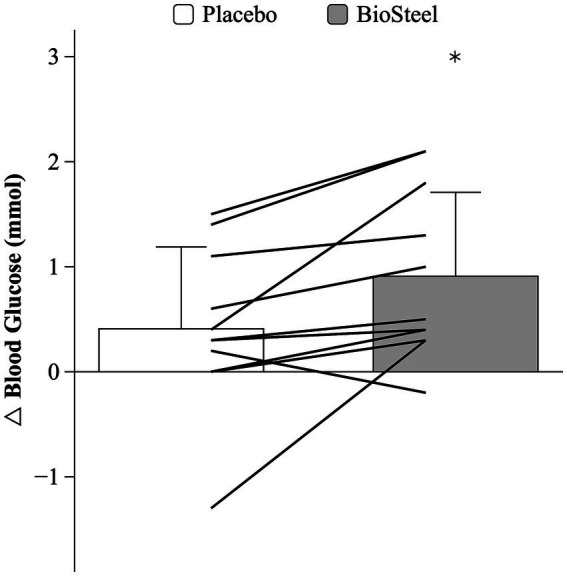
Absolute change in blood glucose for PLA and B-HPSD. Lines denote individual data. *Significantly different than PLA (*p* = 0.016).

There was an interaction between DRINK and TIME on blood glucose during the MSE protocol (*F* = 8.4; *p* = 0.016). Pre-exercise blood glucose was similar between conditions. Blood glucose increased post-exercise with B-HPSD (*p* = 0.016) but not PLA (*p* = 0.333). Post-exercise blood glucose levels were higher following B-HPSD compared to PLA (*p* = 0.032; Figure 3). Participants experienced a significantly greater absolute increase in blood glucose (pre to post) when consuming B-HPSD (0.9 ± 0.8 mmol) compared to PLA (0.4 ± 0.8 mmol), *t*(10) = −2.896, *p* = 0.016 (Figure 4).

### 3.4 Modified Flanker Task

No main (DRINK, *p* = 0.553; TIME, *p* = 0.173) or interaction (DRINK x TIME; *p* = 0.380) effects were detected for accuracy during the modified Flanker Task. Task accuracy remained above 97% for all conditions throughout the testing protocol.

There was a main effect of TIME on mean RT for the modified Flanker Task (*F* = 10.1; *p* = 0.010). Mean RT significantly decreased post exercise across experimental conditions, but the change in RT was similar between DRINKS (Table 7).

**Table 7:** Change in mean response time (ms) for modified Flanker Task pre-and post-MSE.

	Pre	Post*
Placebo	426 ± 35	413 ± 36
B-HPSD	435 ± 40	416 ± 37

*Significantly different than Pre (*p* = 0.01).

## 4 Discussion

The purpose of this investigation was to assess the effectiveness of a single serving of B-HPSD, a commercially available BCAA and VitB-6 containing supplement, on MSE performance during cycling. The results revealed that acute B-HPSD consumption did not alleviate the decline in PO over successive sprint efforts compared to PLA. Additionally, B-HPSD did not influence the exercise-induced changes to HR, RPE, cognitive function, and blood lactate during the trials. In contrast, B-HPSD amplified the rise in blood glucose concentration after exercise.

### 4.1 Does B-HPSD have ergogenic benefits for MSE?

The scientific literature investigating the ergogenic effects of B-HPSD on MSE is limited, making direct comparisons with previous research difficult. In this study, ingesting 7 g of B-HPSD, containing 2,256 mg (29 ± 4 mg/kg BW, Range 23–38 mg/kg BW) of BCAA, did not improve MSE performance. This finding corroborates previous research illustrating that BCAA supplementation did not influence MSE (36). However, methodological differences between these studies, such as exercise type and supplement dose, may have influenced the effectives of the BCAA treatment. First, the exercise protocol used by Pitkänen et al. (36) consisted of repeated 20 s sprints on a treadmill with 100 s recovery intervals, while gradually increasing the speed until exhaustion. In contrast, participants in this investigation completed five 1 km all-out cycling trials separated by 2 min of active recovery. As one protocol utilized a series of maximal efforts and the other utilized incremental exercise, the factors leading to fatigue may have varied. Second, there is a dose-dependent relationship between BCAA ingestion and NH_3_ production (45). Hyperammonaemia, which can occur with high BCAA consumption (46), may have deleterious effects on exercise performance (47). Although, blood NH_3_ was not measured in either investigation, the higher dose of BCAA administered by Pitkänen et al. (36) (200 mg/kg BW) may have led to elevated NH_3_ production, counteracting potential ergogenic effects of BCAA (48). However, some have challenged the relationship between NH_3_ accumulation and the decline in physical performance during exercise (49). Nevertheless, the amount of BCAA administered in the current investigation was lower compared to the dosages used in other studies (29–31, 33–35), and may have been insufficient to elicit an ergogenic benefit.

In contrast, conflicting results have been reported by others where supplementation with BCAA mitigated the decline in MSE during 8 × 50 m swimming sprints with a 3 min recovery period between efforts (16). This disparity may be attributed to several factors associated with variations in supplement composition, as Hsueh et al. (16) administered a larger dosage of BCAA (85 mg/kg BW) and investigated the combined effects of BCAA with ARG and CIT. Previous research has demonstrated that ARG supplementation reduced exercise-related accumulations of NH_3_ (50, 51), while combining CIT and ARG increased plasma ARG concentration more than consuming either amino acid individually (52). These findings suggest that the ergogenic benefits of BCAA may be enhanced when consumed with ARG and CIT, by counteracting the inhibitory effects of hyperammonemia. However, Hsueh et al. (16) did not include a BCAA-only trial to confirm whether BCAA consumption altered NH_3_ accumulation and whether the addition of ARG and CIT influenced NH_3_ metabolism. Additionally, several other studies have examined the effects of BCAA administered with ARG (53, 54) or with ARG and CIT (55, 56) on simulated sport performance over consecutive days/matches. Collectively, these studies demonstrated that supplementation with BCAA combined with ARG and/or CIT improved performance on the final day/match of simulated competition, but not on the first. Since the current study investigated the effects of B-HPSD on a single session of MSE, it remains unknown whether this sport drink would provide ergogenic benefits over consecutive days or bouts of MSE.

Apart from BCAA and VitB, B-HPSD also contains 458 mg glycine, 970 mg glutamine, and 135 mg taurine. While limited research suggests that glutamine (57) and taurine (58) may benefit MSE, further studies are needed to confirm the validity of these claims. Nevertheless, considering the small amount of these amino acids in a single serving of B-HPSD, it is unlikely that they provided significant ergogenic benefits in this investigation.

The results of this study demonstrate that consuming a single serving of B-HPSD does not improve MSE performance. However, further research is required to investigate whether chronic ingestion or acute consumption of higher doses would have ergogenic benefits.

### 4.2 Effects of B-HPSD on blood glucose

In this study, eight out of 11 participants experienced an increase in blood glucose following the PLA trial, although these results were not statistically significant. This is in line with previous research demonstrating an increase in blood glucose after MSE (59). MSE elevates plasma concentrations of glucagon and catecholamines (59), which in turn increase intracellular levels of cyclic AMP and stimulate hepatic glucose production by glycogenolysis and gluconeogenesis (60). This glucose is released into the bloodstream for uptake by active tissues. Changes in blood glucose occur when glucose production and utilization are mismatched. Elevated rates of hepatic glucose mobilization may increase blood glucose concentration, particularly when active muscles are transitioning away from anaerobic glycolysis/glycogenolysis to non-glycolytic fuel sources with successive sprint efforts (17, 19).

The novel finding of this study is that B-HPSD consumption increased post-MSE blood glucose compared to PLA. Although the current study did not specifically investigate glucose metabolism, several mechanisms may explain the observed difference in blood glucose between conditions. First, Blomstrand and Saltin (61) demonstrated that BCAA supplementation increased plasma glucose levels following a 60 min bout of continuous exercise at ~75% V̇O_2max_. Although they suggested that the oxidation of exogenous BCAA elevated concentrations of BCAA-derived acetyl-coenzyme A (acetyl-CoA) that subsequently inhibited pyruvate dehydrogenase (PDH), more recent research has demonstrated that increased BCAA concentrations directly supress PDH activity (62). PDH is the rate-limiting enzyme involved in glucose oxidation, linking glycolysis to the tricarboxylic acid (TCA) cycle by converting pyruvate into acetyl-CoA (63) and is a key aerobic enzyme that drives oxidative phosphorylation with successive sprint efforts (17). Consequently, if the consumption of B-HPSD raised tissue BCAA levels or increased the production of BCAA-derived by-products that inhibited PDH, glucose oxidation could have been reduced, leading to elevated blood glucose levels.

Second, elevated levels of plasma BCAA have been implicated in the development of insulin resistance (64–66). Insulin and BCAA both activate mammalian target of rapamycin complex 1 (mTORC1) (67), a regulator of glucose metabolism (68). In normal conditions, insulin initiates the activation cascade by binding to its receptor on the cell surface, leading to the phosphorylation of insulin receptor substrate 1 (IRS-1) at multiple tyrosine residues. This activates phosphoinositide 3-kinase (PI3K) and subsequently leads to Akt phosphorylation (67). Akt orchestrates various downstream effects, including the phosphorylation and inhibition of the tuberous sclerosis complex 1/2 (TSC 1/2) lifting its suppression on mTORC1, and the translocation of glucose transporter 4 (GLUT4) to the cell membrane increasing glucose uptake (69). Increased plasma BCAA levels also activate mTOR independently of TSC regulation (70). Acute infusion of an amino acid mixture in young, healthy subjects increased plasma BCAA concentration 2-fold and amplified insulin-induced serine phosphorylation of IRS-1 in skeletal muscle (71). This phosphorylation pattern hindered the typical insulin-triggered tyrosine phosphorylation of IRS-1, impeding the translocation of GLUT4 to the cell membrane, elevating blood glucose levels. However, when Everman et al. (72), infused a BCAA-only mixture that increased plasma BCAA concentration more than 2.5-fold, glucose uptake was maintained. Collectively, these studies suggest that increased concentrations of plasma amino acids, but not BCAA, led to decreased insulin sensitivity. Although plasma amino acid concentrations were not measured in this investigation, the total amount of amino acids and BCAA administered in this study (~ 49 ± 6 mg/kg BW; Range 39–64 mg/kg BW) was much lower when compared to Tremblay et al. (71), (990 mg/kg BW). This lower dosage likely did not elevate plasma amino acid levels to the same extent, and consequently, would not have led to comparable levels of insulin resistance.

Third, increasing the bioavailability of BCAA through exogenous sources enhances the production of gluconeogenic precursors like alanine (46). Alanine is formed when glutamate, from the transamination of BCAA, combines with pyruvate in the presence of alanine aminotransferase. Alanine is transported to hepatocytes, where it is converted into pyruvate and subsequently undergoes gluconeogenesis to form glucose. This newly formed glucose is then released into the bloodstream to maintain blood glucose homeostasis during exercise (73). However, if BCAA-induced stimulation of the glucose-alanine cycle coincides with reductions in tissue glucose utilization, as observed in MSE (1, 17), blood glucose levels would increase. Consequently, if oxidation of the exogenous BCAA found in B-HPSD produced a sufficient quantity of gluconeogenic precursors to stimulate the glucose-alanine cycle, it may have contributed to the elevation in blood glucose.

While tempting to consider these mechanisms as explanations for the disparity in blood glucose between conditions, further research is required to confirm if a single serving of B-HPSD would drive such metabolic changes.

### 4.3 Effects of B-HPSD on cognitive function

The results of this study demonstrate that MSE had a positive effect on cognitive function 15 min after exercise, corroborating previous research (74). However, these findings contradict research illustrating that BCAA improved cognitive performance following sustained exercise (30, 31), and after a repeated reactive agility test (75). Methodological differences may explain these contrary findings. The impact of exercise on cognitive function is dependent on the interaction between exercise intensity and the time interval between exercise and cognitive testing. With respect to intense exercise, research suggests that the greatest effect on cognitive performance is observed 11–20 min following the activity (76). In the current investigation, the modified Flanker Task was administered 15 min after the completion of the MSE protocol, presumably when the effect of exercise on cognitive performance was amplified. Previous research measured cognitive function immediately after (75), within 10 min (31), and 1–2 h post-exercise (30), likely when the effect of exercise on cognitive performance was subdued. The exact mechanism behind this opposing result remains uncertain. However, it is plausible to speculate that when cognitive assessments are conducted 11–20 min after MSE, the intensified effect of exercise on cognitive function may overshadow the potential benefits of BCAA. Nevertheless, further research is needed to confirm such a proposition.

### 4.4 Limitations

One of the limitations of this study was the participants’ diet prior to testing. Participants were asked to maintain an identical nutritional intake for 24 h before each of the two experimental trials, which was verbally confirmed. However, food recall data was not collected nor was the macronutrient and energy content controlled between participants. Previous research investigating the effects of BCAA on exercise performance have required participants to undergo glycogen depletion (30) or an overnight fast (77) before BCAA ingestion. The dietary controls of the current study allowed for a pragmatic investigation into the effects of B-HPSD as participants exercised in a fed state, which is common practice leading into athletic competition. Nevertheless, it is important to recognize that inter-individual differences in macronutrient content and energy intake may have influenced substrate utilization and exercise performance.

Another limitation was the small number of ‘all-out’ sprints as most team sports are played over the course of hours, not minutes. Extending these findings to team sport performance, repeated sessions of MSE, or other types of prolonged, intermittent high-intensity exercise is difficult. Whether B-HPSD would have an ergogenic effect on other forms of exercise requires further investigation.

## 5 Conclusion

In conclusion, this investigation demonstrates that a single serving of B-HPSD did not provide an ergogenic benefit to MSE performance when consumed 30 min before exercise. However, the increase in post-exercise blood glucose was amplified with B-HPSD when compared to PLA, which may warrant future investigations into the effects of this product on exercise that threatens blood glucose homeostasis.

## Data availability statement

The original contributions presented in the study are included in the article/supplementary material, further inquiries can be directed to the corresponding author.

## Ethics statement

The studies involving humans were approved by Sheridan Research Ethics Board. The studies were conducted in accordance with the local legislation and institutional requirements. The participants provided their written informed consent to participate in this study.

## Author contributions

SF: Conceptualization, Data curation, Formal analysis, Funding acquisition, Investigation, Methodology, Project administration, Resources, Supervision, Validation, Visualization, Writing – original draft, Writing – review & editing.
